# Textural features on ^18^F-FDG PET/CT and dynamic contrast-enhanced MR imaging for predicting treatment response and survival of patients with hypopharyngeal carcinoma

**DOI:** 10.1097/MD.0000000000016608

**Published:** 2019-08-16

**Authors:** Chih-Kai Wong, Sheng-Chieh Chan, Shu-Hang Ng, Chia-Hsun Hsieh, Nai-Ming Cheng, Tzu-Chen Yen, Chun-Ta Liao

**Affiliations:** a School of Medicine, Chang Gung University, Taoyuan; b Department of Nuclear Medicine, Hualien Tzu Chi Hospital, Buddhist Tzu Chi Medical Foundation, Hualien; c Department of Diagnostic Radiology; d Division of Medical Oncology, Department of Internal Medicine, Linkou Chang Gung Memorial Hospital and Chang Gung University, Taoyuan; e Department of Nuclear Medicine, Keelung Chang Gung Memorial Hospital, Keelung; f Department of Nuclear Medicine; g Department of Otorhinolaryngology, Linkou Chang Gung Memorial Hospital and Chang Gung University, Taoyuan, Taiwan.

**Keywords:** hypopharyngeal carcinoma, MRI, PET/CT, prognosis, texture analysis

## Abstract

The utility of multimodality molecular imaging for predicting treatment response and survival of patients with hypopharyngeal carcinoma remains unclear. Here, we sought to investigate whether the combination of different molecular imaging parameters may improve outcome prediction in this patient group.

Patients with pathologically proven hypopharyngeal carcinoma scheduled to undergo chemoradiotherapy (CRT) were deemed eligible. Besides clinical data, parameters obtained from pretreatment 2-deoxy-2-[fluorine-18]fluoro-D-glucose positron emission tomography/computed tomography (^18^F-FDG PET/CT), dynamic contrast-enhanced (DCE) magnetic resonance imaging (MRI), and diffusion-weighted MRI were analyzed in relation to treatment response, recurrence-free survival (RFS), and overall survival (OS).

A total of 61 patients with advanced-stage disease were examined. After CRT, 36% of the patients did not achieve a complete response. Total lesion glycolysis (TLG) and texture feature entropy were found to predict treatment response. The transfer constant (K^*trans*^), TLG, and entropy were associated with RFS, whereas K^*trans*^, blood plasma volume (V_*p*_), standardized uptake value (SUV), and entropy were predictors of OS. Different scoring systems based on the sum of PET- or MRI-derived prognosticators enabled patient stratification into distinct prognostic groups (*P* <.0001). The complete response rate of patients with a score of 2 was significantly lower than those of patients with a score 1 or 0 (14.7% vs 58.9% vs 75.7%, respectively, *P* = .007, respectively). The combination of PET- and DCE-MRI-derived independent risk factors allowed a better survival stratification than the TNM staging system (*P* <.0001 vs .691, respectively).

Texture features on ^18^F-FDG PET/CT and DCE-MRI are clinically useful to predict treatment response and survival in patients with hypopharyngeal carcinoma. Their combined use in prognostic scoring systems may help these patients benefit from tailored treatment and obtain better oncological results.

## Abstract 2

Supplemental Digital Content is available in the text

## 1 Introduction

Hypopharyngeal carcinoma tends to be aggressive and comes with a high fatality rate. Approximately 80% of patients have stage III to IV disease at the time of diagnosis.^[1]^ The mainstay of treatment consists of surgery and radiotherapy, either with or without chemotherapy. In recent years, the use of chemoradiotherapy (CRT) in patients with locally advanced hypopharyngeal carcinoma has gained momentum, with treatment outcomes being comparable with those of surgery.^[2]^ However, the current 5-year survival rate for patients with hypopharyngeal carcinoma (30%) remains suboptimal,^[2,3]^ with 20% of patients still having residual disease following treatment with curative intent.^[4]^ Owing to the advanced disease stages at presentation and the high treatment failure rates, an improved prognostic stratification of patients with hypopharyngeal carcinoma would be paramount.^[4,5]^

Magnetic resonance imaging (MRI) has been extensively used as an anatomic imaging modality in patients with head and neck squamous cell carcinoma (HNSCC). Dynamic contrast-enhanced MRI (DCE-MRI) and diffusion-weighted MRI (DWI) are functional imaging techniques that are increasingly being implemented in conventional MRI for investigating intrinsic tumor characteristics.^[6,7,8,9]^ DCE-MRI allows measuring tumor microvascularity—which has prognostic significance in HNSCC.^[10,11,12,13,14]^ DWI—which measures tumor cellularity—has been also shown to predict treatment response in patients with HNSCC.^[15]^

The 2-deoxy-2-[fluorine-18]fluoro-D-glucose positron emission tomography/computed tomography (^18^F-FDG PET/CT) has been extensively applied in the field of oncology. Moreover, PET-derived metabolic parameters—including standardized uptake value (SUV), metabolic tumor volume (MTV), and total lesion glycolysis (TLG)—are clinically useful to predict prognosis in patients with HNSCC.^[16,17,18,19,20,21]^ MTV is a measure of FDG-avid disease volume, whereas TLG further incorporates the intensity of FDG uptake at the lesion site. Growing evidence indicates that texture features or heterogeneity on PET images can potentially be useful for predicting survival in patients with HNSCC.^[22,23,24]^

The integration of functional information from DWI, DCE-MRI, and ^18^F-FDG PET/CT holds promise for improving the prognostic stratification of patients with malignancies. We have previously shown that the combination of clinical variables and PET/CT or functional MRI parameters refines prognosis prediction in patients with pharyngeal carcinoma.^[13,14,22,25]^ However, these studies included both oropharyngeal and hypopharyngeal cancers and did not report the utility of imaging parameters for predicting treatment response. Currently, oropharyngeal and hypopharyngeal cancers are classified separately because the staging system and the management strategies for patients with oropharyngeal cancer accompanied by human papillomavirus infection differ from those for patients with hypopharyngeal carcinoma.^[26]^ In addition, the presence of residual tumor after CRT is not uncommon for patients with hypopharyngeal cancer ^[4]^ and poses significant challenges to clinical management. Unfortunately, robust pretreatment molecular markers for predicting treatment response in patients with hypopharyngeal cancer have not yet been identified. Under these circumstances, we designed the present study to investigate the clinical value of multimodality imaging parameters from ^18^F-FDG PET/CT, DCE-MRI, and DWI in the prediction of treatment response and survival in CRT-treated patients with hypopharyngeal carcinoma.

## 2 Materials and methods

### 2.1 Patients

Consecutive patients with newly diagnosed hypopharyngeal carcinoma who were scheduled for chemoradiotherapy with curative intent were deemed eligible. Ethics approval was granted by the institutional review board of our hospital (protocol no. 98–3582B) and the study complied with the tenets of the Declaration of Helsinki. The inclusion criteria were as follows:

(1) biopsy-proven hypopharyngeal carcinoma,
(2) absence of distant metastases, and
(3) no contraindications to MRI or ^18^F-FDG PET/CT.

Patients with a history of previous head and/or neck malignancies, concomitant cancers in different anatomical districts, or renal failure were excluded.

### 2.2 Pretreatment ^18^F-FDG PET/CT and MRI studies

^18^F-FDG PET/CT and MRI were performed within 2 weeks of each other before definitive treatment. The details of the scanning protocols have been described in a previous study.^[13]^ Before undergoing ^18^F-FDG PET/CT imaging, the study patients fasted for at least 6 hours and all of them had glucose concentrations less than 150 mg/dL. Scans were performed with a PET/CT system (Discovery ST 16; GE Healthcare, Milwaukee, WI) consisting of a PET scanner and a 16-section CT scanner. We obtained PET emission images between 50 and 70 min after injection of ^18^F-FDG (370 MBq) in the 2-dimensional mode, with a 3-min scanning time per table position. Before PET acquisition, a standardized helical CT scan was acquired from the head to the proximal thigh using the following settings: transverse 3.0-mm collimation × 16 modes, 100 kVp, 100 mAs, 0.5-s tube rotation, 35-mm/s table speed, and 1.5 pitch. No intravenous iodinated contrast agent was used. We resized CT data from a 512 × 512 matrix to a 128 × 128 matrix to match PET results and generate fused images and CT-based transmission maps. We reconstructed PET images with CT for attenuation correction and an ordered-subset expectation maximization iterative reconstruction algorithm (4 iterations and 10 subsets).

A 3-T MRI scanner (Magnetom Trio with TIM, Siemens, Erlangen, Germany) was used in this study. Conventional MRI images of the head and neck region were obtained in the axial and coronal projections using the following sequences: T1-weighted turbo spin echo TSE; T2-weighted TSE with fat saturation; and postcontrast fat-saturated T1-weighted TSE. Transverse images were obtained at a 5-mm section thickness. A dedicated neck coil was used. DWI was obtained using single shot spin-echo echo-planar imaging with a modified Stejskal–Tanner diffusion gradient pulsing scheme. Motion-probing gradients (b-value = 800 s/mm^2^) were applied along the 3 orthogonal directions. Imaging slices and coverage were identical for both T1- and T2-weighted images. The repetition time (TR) and echo time (TE) were 8200 ms and 84 ms, respectively. DCE-MRI was acquired using a 3D T1-weighted spoiled gradient-echo sequence with the following parameters: TR/TE = 3.5/1.13 ms, 230 × 230-mm field of view, and 108 × 128 matrix. The same imaging slice and coverage of conventional T1- and T2-weighted images were applied. To minimize the inflow effect from carotid arteries, a spatial saturation slab was implanted below the acquired region. Baseline longitudinal relaxation time (T1_0_) values were calculated from images acquired with different flip angles (4°, 8°, 15°, and 25°) before contrast agent injection. The dynamic series was based on the use of the same sequence with a 15° flip angle. After 4 acquisitions of the dynamic baseline scanning, a standard dose (0.1 mmol/kg body weight) of gadopentetate dimeglumine (Gd-DTPA; Magnevist, Bayer-Schering, Burgess Hill, UK) was administered by a power injector through a cannula placed in the antecubital vein (rate = 3 mL/s) and immediately followed by a saline flush. A total of 80 volumes were acquired (temporal resolution = 3.3 s).

### 2.3 Treatment and follow-up schedule

Patients were treated with intensity-modulated radiotherapy using 6-MV photon beams at 2 Grays (Gy) per fraction, with 5 fractions per week. Radiation therapy was delivered at a dose of 46 Gy to the gross tumor area (with at least 1-cm margins) and the entire neck, followed by a cone-down boost at 72 Gy to the gross tumor area and close margins. All participants received intensity-modulated radiotherapy. Concurrent chemotherapy (CCRT) was administered as follows: intravenous cisplatin 50 mg/m^2^ on day 1, and oral tegafur 800 mg/day plus oral leucovorin 60 mg/day from days 1 to 14. This regimen was repeated every 2 weeks throughout the course of radiotherapy.^[27]^

Patients received routine clinical follow-up examinations every 1 to 3 months. Follow-up head-neck MRI was performed 3 months after treatment completion. Subsequently, an additional MRI or CT scan was performed every 6 months or in presence of suspected clinical recurrences. Patients were followed for at least 24 months after treatment completion or until death.

### 2.4 Image analysis

The apparent diffusion coefficient (ADC) maps were reconstructed on a pixel-by-pixel basis using software integral to the MRI unit. The ADC values were measured on ADC maps by an experienced head and neck radiologist by drawing the region-of-interest (ROI) on the primary tumor. DCE-MRI analyses were performed using MATLAB 7.0 (The Mathworks, Natick, MA). The extended Kety model was used in a voxel-wise manner for pharmacokinetic analysis.^[28]^ The arterial input function was extracted using the blind source separation algorithm.^[29]^ The following pharmacokinetic parameters were collected: volume transfer constant (K^*trans*^), relative extravascular extracellular space (V_*e*_), relative vascular plasma volume (V_*p*_), and efflux rate constant (K_*ep*_).

Tumor segmentation on the PET images was performed with the PMOD 3.2 software package (PMOD Technologies Ltd., Zurich, Switzerland). First, an experienced nuclear medicine physician drew boundaries large enough to include the primary tumor on PET scans. Second, the tumor boundaries were defined using a fixed SUV threshold of 2.5. Finally, SUV (maximum; SUVmax), MTV, and TLG of the lesion were automatically calculated by the software as previously described.^[22]^

The texture features or heterogeneity parameters of PET images were analyzed using the normalized gray-level co-occurrence matrix (NGLCM) and the neighborhood gray-tone difference matrix (NGTDM). Calculations were performed as previously described.^[23]^ Second-order parameters were calculated using the NGLCM to obtain the uniformity, entropy, dissimilarity, homogeneity, and inverse different moment values. Higher-order parameters were calculated using NGTDM to obtain coarseness, contrast, busyness, and complexity. These parameters were calculated using an in-house software package (Chang-Gung Image Texture Analysis toolbox, CGITA) implemented with MATLAB 2012a (Mathworks Inc., Natick, MA).

### 2.5 Outcome determination and statistical analysis

The treatment response after definitive CCRT, overall survival (OS), and recurrence-free survival (RFS) served as the main outcome measures. The comprehensive treatment response to CCRT was graded according to the Response Evaluation Criteria in Solid Tumors [RECIST], version 1.1.^[30]^ A receiver operating characteristic curve analysis was used to calculate the cutoff values for the variables related to treatment response. A Pearson's chi-square test was conducted to identify risk factors for response. Logistic regression models were subsequently constructed to identify the independent predictors of treatment response. OS was calculated from the date of diagnosis to the date of death, or to the date of the last follow-up examination for surviving patients. RFS was defined as the time between the end of treatment and the date of recurrence (i.e., tumor relapse or death), or the date of the last follow-up examination. Survival curves were plotted using the Kaplan–Meier method. The effect of each individual variable was initially evaluated using univariate analysis. Cox regression models were used to identify the independent predictors of survival. Based on the results of the multivariate regression analyses, we devised 2 distinct scoring systems for predicting treatment response and OS/DFS, respectively. The systems were based on summation of the independent predictors identified in multivariate analysis—with each variable assigned a score of 1 (if present) or 0 (if absent). The total score reflected the number of independent risk factors identified in each patient (i.e., a participant with a score of 2 carried 2 independent adverse risk factors). Two-tailed *P* values <.05 were considered statistically significant.

## 3 Results

### 3.1 Study participants

Between January 2010 and June 2013, we identified 72 patients with hypopharyngeal cancer who were potentially eligible for the study (Table 1). However, patients with unsatisfactory DWI or DCE-MRI images (n = 9) and lost to follow-up (n = 2) were excluded. Consequently, the final study cohort consisted of 61 patients. At the time of analysis, 22 (36.1%) patients were dead and 32 experienced tumor recurrence. The median follow-up time for all patients was 3 years. The 3-year RFS and OS rates were 52.4% and 63.9%, respectively. Twenty-two patients did not achieve complete remission after CRT, among whom 16 had locoregional failures, 3 had distant failures, and 3 had both locoregional plus distant failures. The distribution of the MRI functional parameters, conventional PET parameters, and PET heterogeneity parameters are summarized in Supplementary Table 1.

**Table 1:**
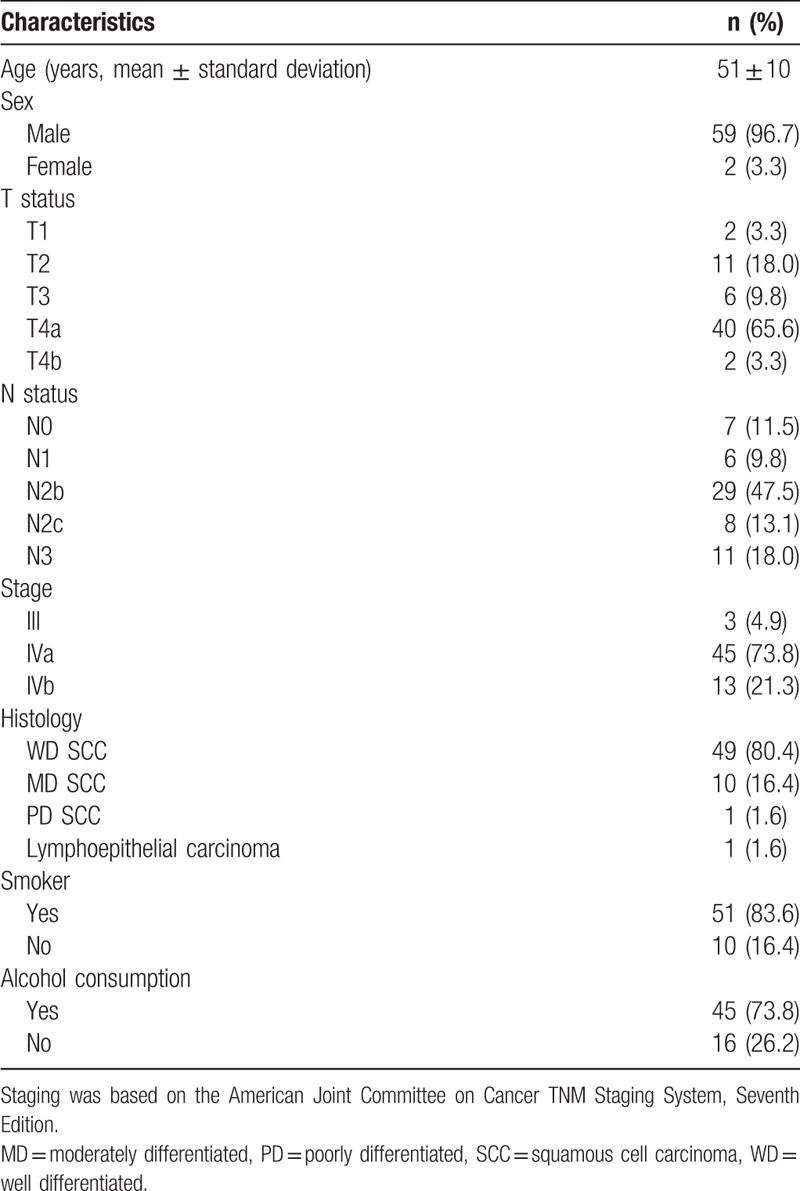
General characteristics of the study participants (n = 61).

Data on p16 immunohistochemistry were available for 15 patients. Positive and negative p16 immunostaining was identified in 2 and 13 cases, respectively. In the former group, the percentage of p16 positivity was 5% and 60%, respectively.

### 3.2 Association of clinical and imaging parameters with treatment response

**Figure 1:**
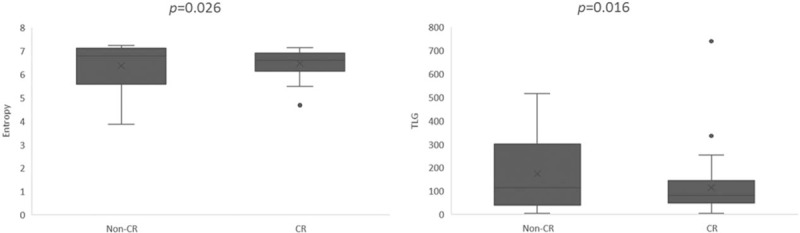
Box-whisker plot showing the value of TLG and texture feature entropy on PET for predicting treatment response in patients with primary hypopharyngeal carcinoma treated with chemoradiotherapy. CRs showed significantly lower entropy and TLG values compared with non-complete responders (non-CRs). CRs = complete responders, PET =  positron emission tomography, TLG = total lesion glycolysis.

After definitive CCRT, 22 patients (36%) did not achieve a complete response to treatment and were defined as non-complete responders. Table 2 shows the relations between response to CCRT and different clinical and imaging parameters. PET-derived MTV, TLG, and texture feature entropy differed significantly between complete and non-complete responders (*P* <.05; Fig. 1). Another PET texture feature, uniformity, exhibited a borderline association with response to CCRT (*P* = .079). Other variables did not differ significantly between complete and non-complete responders. The dose of radiation therapy delivered to the gross tumor did not reach 72 Gy in 3 patients, and 2 of them did not achieve complete response. Of the 59 patients who received a full therapeutic dose, 19 were non-complete responders. There were no significant intergroup differences in terms of response rate (*P* = .27).

**Table 2:**
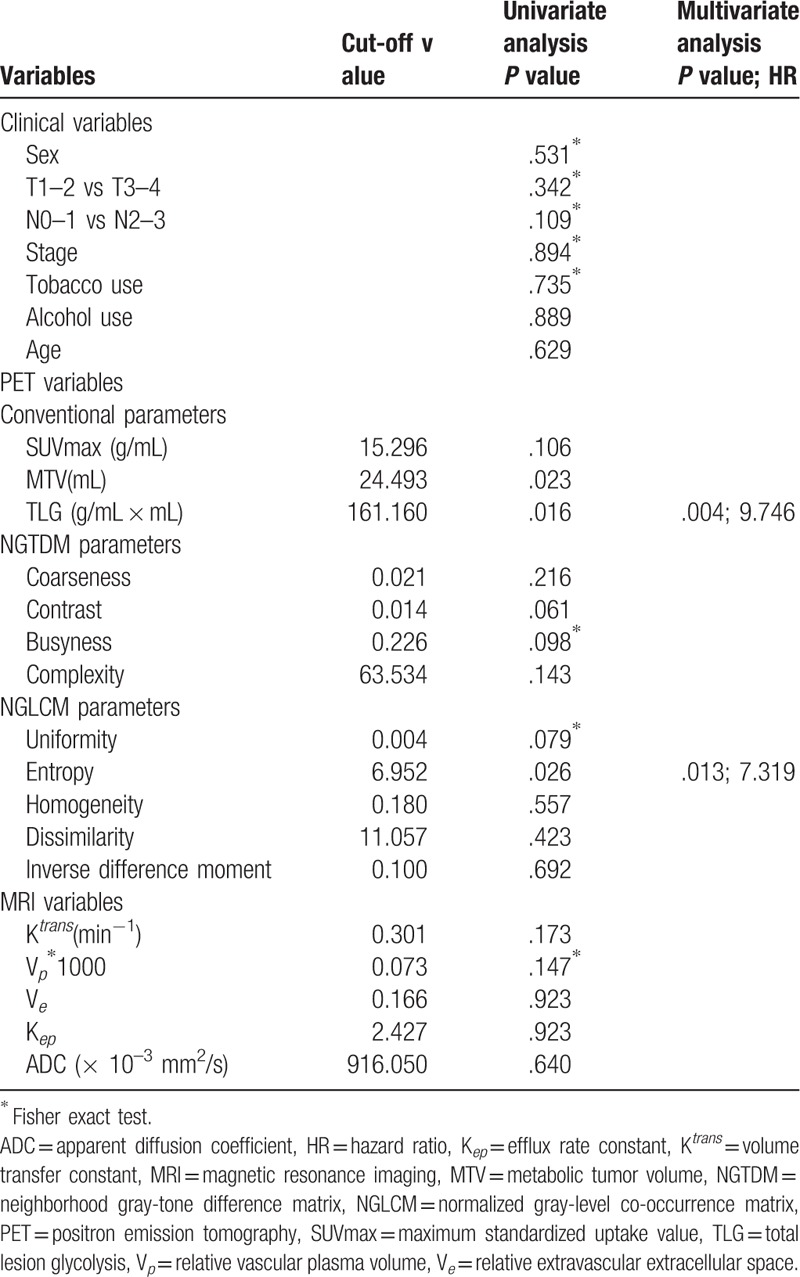
Predictors of treatment response in patients with primary hypopharyngeal carcinoma treated with chemoradiotherapy.

In logistic regression analysis, high TLG (*P* = .004, hazard ratio [HR] = 9.746) and entropy (*P* = .013, HR = 7.319) were the only independent predictors of non-complete response.

**Figure 2:**
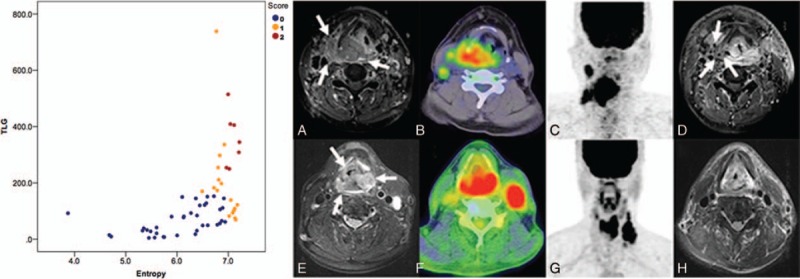
Scatter plot depicting the distribution of pretreatment texture feature entropy values against TLG values, categorized by the scoring system. Representative PET and MRI images for cases with high (red color) or low (blue color) scores are also shown. (a) A patient with stage T4aN2b hypopharyngeal squamous cell carcinoma (arrow) and a score of 2 based on the treatment response scoring system devised in the present study. (b) An ^18^F-FDG PET/CT image depicting the primary tumor, which had an entropy value of 7.11 and (c) a TLG value of 405.21 g/mL × mL. (d) After chemoradiotherapy, the patient still had a residual tumor (arrow) at the locoregional site. (e) An ^18^F-FDG PET/CT image of a patient with stage T4aN3 hypopharyngeal squamous cell carcinoma (arrow) and a score of 0. (f) The ^18^F-FDG PET/CT image had an entropy value of 6.89 and (g) a TLG value of 110.97 g/mL × mL. (h) The patient achieved complete remission after definitive treatment. CT = computed tomography, MRI = magnetic resonance imaging, PET = positron emission tomography, TLG = total lesion glycolysis.

We subsequently devised a scoring system for predicting treatment response based on the number of independent risk factors identified in multivariate analysis. The presence or absence of each risk factor was assigned a score of 1 and 0, respectively, resulting in scores ranging from 0 to 2. This system allowed patient stratification into distinct risk groups characterized by different responses to treatment (Table 3). Compared with patients with a score of 0 (reference category), those with a score of 1 or 2 had significant poorer complete response rates (HR = 2.178, *P* = .007; HR = 18.667, *P* = .003, respectively). The complete response rate of patients with a score of 2 was significantly lower than those of cases with a score 1 or 0, (14.7% vs 58.9% vs75.7%, respectively, *P* = .007). Figure 2 illustrates the utility of the scoring system for stratifying response to treatment.

**Table 3:**
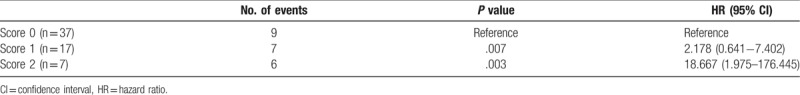
Comparison of treatment responses after chemoradiotherapy according to the prognostic scoring system.

### 3.3 Predictors for survivals

**Figure 3:**
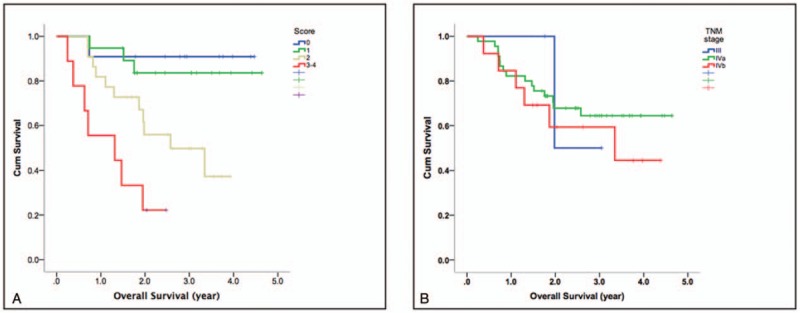
Kaplan–Meier plots of overall survival in patients with primary hypopharyngeal carcinoma stratified acco rding to our prognostic scoring system (a) and the TNM Staging System (b). The combination of pretreatment texture feature entropy and DCE-MRI parameters enabled a better prognostic stratification than the TNM Staging System (*P* <.0001 vs .691, respectively). DCE-MRI = dynamic contrast-enhanced MRI.

Univariate analysis identified the following parameters as significant predictors of RFS: K^*trans*^ (*P* = .046), V_*e*_ (*P* = .035), MTV (*P* <.001), TLG (*P* = .01), and entropy (*P* = 0.002). After adjustment for confounders in multivariate analysis, only K^*trans*^, TLG, and entropy (*P* = 0.009) were retained in the model as adverse prognostic factors (Table 4). The results of univariate analysis also revealed that K^*trans*^ (*P* = .01), V_*p*_ (*P* = .025), V_*e*_ (*P* = .028), ADC (*P* = .016), SUVmax (*P* = .025), TLG (*P* = .048), and texture feature entropy (*P* = .025) were significantly associated with OS. After allowance for potential confounders in multivariate analysis, only the K^*trans*^, V_*p*_, SUVmax, and entropy were identified as independent predictors of OS (Table 4). Both T and N status were not significantly associated with RFS or OS in univariate analysis. A scoring system was therefore devised to predict OS and RFS based on DCE-MRI- and PET-derived parameters (Supplementary Table 2). This system was capable of identifying different patient subgroups characterized by distinct OS (*P* <.0001) and RFS (*P* = .001) rates. Notably, the scoring system allowed a better prognostic stratification compared with the current TNM Staging System (Fig. 3). Examples of patients with different prognostic scores are provided in Figure 4 .

**Table 4:**
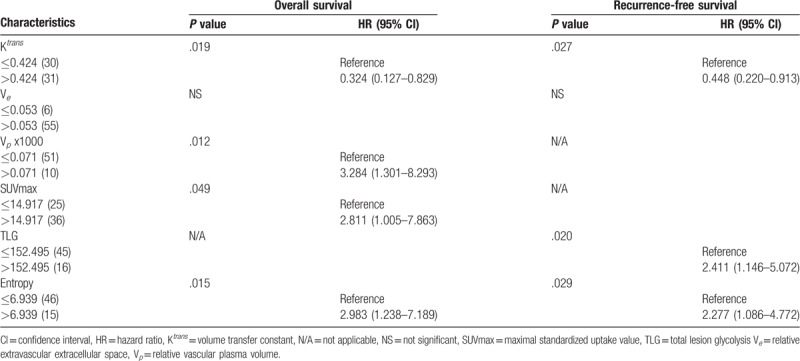
Multivariate analyses of risk factors associated with overall survival and recurrence-free survival rates in patients with primary hypopharyngeal cancer.

**Figure 4:**
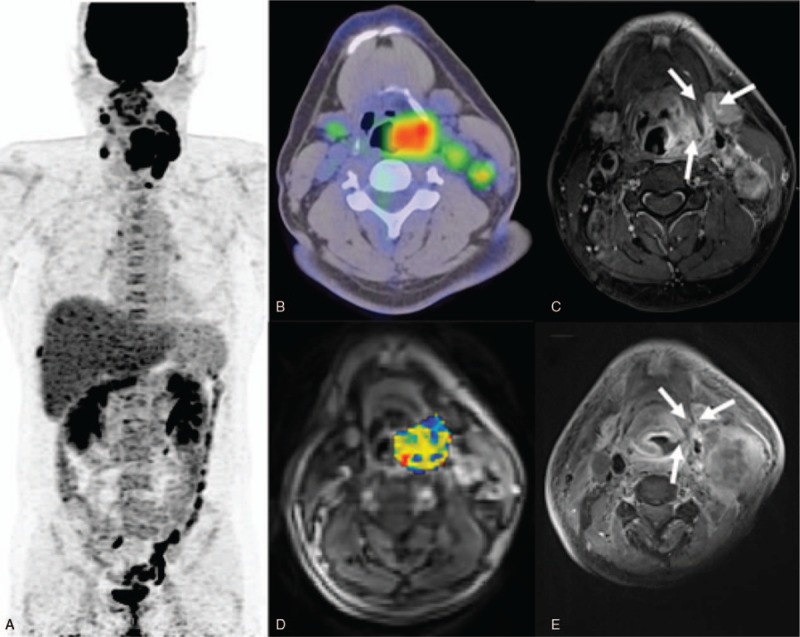
**1.** (a) A PET image of a 40-year-old male patient with stage IVa hypopharyngeal squamous cell carcinoma and a score of 4. (b) The ^18^F-FDG PET/CT image showed a high primary tumor entropy value (7.217). (c) A left hypopharyngeal tumor was also identified on the corresponding axial-enhanced T1-weighted MRI image. (d) A DCE-MRI image with an overlaid volume transfer rate constant (K^*trans*^) map of the primary tumor exhibited a low value of 0.317 min^−1^. (e) The patient had a persistent tumor at the locoregional site (arrow) and died 9 months thereafter. **4–2.** (a) A positron emission tomography (PET) image of a 58-year-old male patient with stage IVb hypopharyngeal squamous cell carcinoma staging and a score of 1. (b) The ^18^F-FDG PET/CT image showed a low entropy value (6.785). (c) The corresponding axial-enhanced T1-weighted MRI also revealed a bulky right hypopharyngeal tumor. (d) The corresponding DCE-MRI with an overlaid volume transfer rate constant (K^*trans*^) map had a high value (0.655 min^−1^). (e) A post-treatment MRI showed complete remission of the primary tumor. After 60 months of follow-up, the patient is still alive without recurrences. CT = computed tomography, DEC = dynamic contrast-enhanced, MRI = magnetic resonance imaging, PET = positron emission tomography.

**Figure 4 (Continued):**
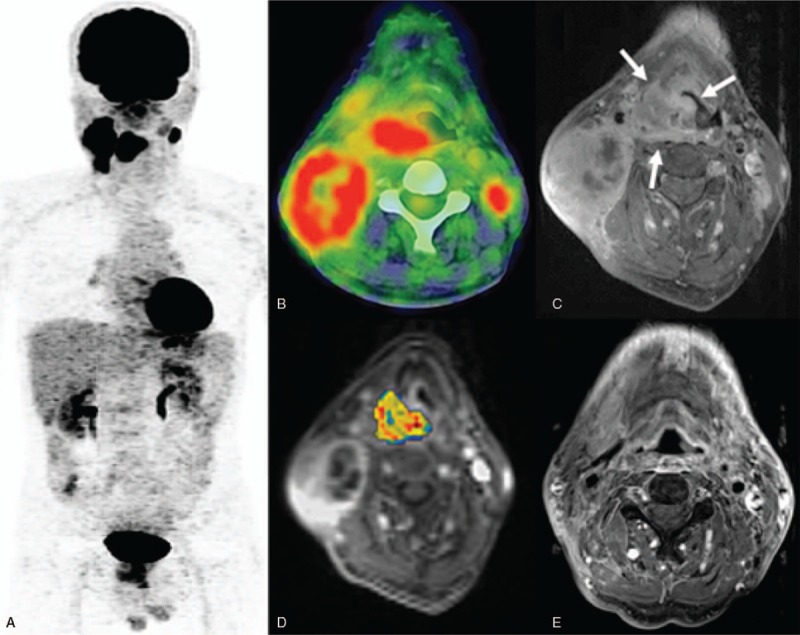
**1.** (a) A PET image of a 40-year-old male patient with stage IVa hypopharyngeal squamous cell carcinoma and a score of 4. (b) The ^18^F-FDG PET/CT image showed a high primary tumor entropy value (7.217). (c) A left hypopharyngeal tumor was also identified on the corresponding axial-enhanced T1-weighted MRI image. (d) A DCE-MRI image with an overlaid volume transfer rate constant (K^*trans*^) map of the primary tumor exhibited a low value of 0.317 min^−1^. (e) The patient had a persistent tumor at the locoregional site (arrow) and died 9 months thereafter. **4–2.** (a) A positron emission tomography (PET) image of a 58-year-old male patient with stage IVb hypopharyngeal squamous cell carcinoma staging and a score of 1. (b) The ^18^F-FDG PET/CT image showed a low entropy value (6.785). (c) The corresponding axial-enhanced T1-weighted MRI also revealed a bulky right hypopharyngeal tumor. (d) The corresponding DCE-MRI with an overlaid volume transfer rate constant (K^*trans*^) map had a high value (0.655 min^−1^). (e) A post-treatment MRI showed complete remission of the primary tumor. After 60 months of follow-up, the patient is still alive without recurrences. CT = computed tomography, DEC = dynamic contrast-enhanced, MRI = magnetic resonance imaging, PET = positron emission tomography.

## 4 Discussion

Residual cancer has been reported in 30% to 35% of patients with hypopharyngeal carcinoma following treatment with curative intent.^[4,31]^ Early prediction of persistent disease is crucial for patients with hypopharyngeal cancer who received CRT because salvage surgery may offer a survival benefit in patients with resectable tumors. In this study, we investigated the impact of pretreatment ^18^F-FDG PET/CT, DCE-MRI, and DWI for predicting treatment response and prognosis in patients with primary hypopharyngeal carcinoma. Our results revealed that PET-derived TLG and the texture feature entropy were reliable predictors of response to CRT. The combination of TLG and entropy may, therefore, allow the identification of patients at high risk of residual cancer.

Growing evidence indicates that metabolic parameters based on pretreatment ^18^F-FDG PET/CT may improve the prognostic stratification of patients with HNSCC.^[32]^ TLG integrates information on tumor volumetric burden and metabolic activity. The results from a meta-analysis have shown that patients with HNSCC patients and high TLG values have a 3.10-fold higher risk of tumor progression/recurrence than those with low TLG values.^[19]^ Lim et al^[33]^ also demonstrated that TLG predicts local recurrence and overall survival in CRT-treated patients with oropharyngeal carcinoma. Besides confirming previous observations, our current data demonstrate that TLG is a valuable predictor (HR: 9.746) of treatment response in patients with hypopharyngeal carcinoma who had undergone CRT.

Intratumoral heterogeneity—an imaging biomarker associated with tumor aggressiveness—may have prognostic significance in patients with malignancies.^[34]^ Recent studies have shown that PET heterogeneity parameters or texture features may be superior to other imaging or clinical parameters in informing prognosis.^[22,25,35,36]^ Oh et al^[24]^ have previously reported patients with hypopharyngeal carcinoma who responded to CRT had lower coarseness (*P* <.001) and busyness (*P* = .015) compared with those who did not. In the present study, entropy—but not coarseness and busyness—was the only significant texture feature associated with treatment response (Table 2). The discrepant findings may be attributed at least in part to distinct treatment strategies and methodological differences in heterogeneity analysis.

We previously explored the utility of pretreatment PET/CT, DCE-MRI, and DWI for predicting tumor recurrence after CRT in patients with oropharyngeal or hypopharyngeal cancer.^[14,25]^ Our results revealed that K^*trans*^ was independently associated with local control, whereas V_*e*_ and ADC were independent predictors of regional control. In the present study, we instead explored the clinical utility of functional imaging parameters for predicting treatment response in patients with hypopharyngeal cancer, with a special emphasis on the detection of residual tumors. Unexpectedly, functional MRI parameters were unable to predict response to CRT. This discrepancy may be explained by the inclusion of PET texture analysis and of a higher number of patients with pure hypopharyngeal carcinoma.

The combined assessment of ^18^F-FDG PET metabolic and heterogeneity parameters can improve the prognostic stratification of patients with hypopharyngeal carcinoma. Here, we devised prognostic scoring systems based on the independent risk factors identified in multivariate analysis. The risk of residual tumors was significantly higher in patients with a score of 2 or 1 (HRs: 18.667 and 2.178, respectively) than in those with a score of 0. Our scoring system may be helpful to allocate patients to the most suitable treatment strategy. Recently, several phase III trials failed to demonstrate a survival advantage for docetaxel, cisplatin, and 5-fluorouracil (TPF)-based induction chemotherapy over standard cisplatin-based concurrent chemoradiotherapy for patients with head and neck carcinoma.^[37,38,39]^ These results led to hypothesize the existence of specific risk factors for treatment failure. Because traditional prognostic factors (e.g., tumor stage) may be insufficient for identifying poor responders to CRT in clinical trials,^[40]^ the efficacy of induction chemotherapy could be underestimated. Patients identified with an unfavorable outcome (e.g., those with a score of 2) may be suitable candidates to receive induction chemotherapy in future clinical trials. Conversely, patients with a favorable prognostic score (e.g., those with a score of 0) may attain a favorable tumor control without resorting to aggressive CRT, ultimately improving quality of life.^[41]^

With regard to multimodal functional imaging for survival assessment, we previously demonstrated that K^*trans*^, K_*ep*_, and alcohol consumption independently predicted RFS, whereas K^*trans*^, K_*ep*_, and uniformity were independent risk factors for OS.^[22]^ After the exclusion of patients with oropharyngeal carcinoma from the present study, multivariate analysis revealed that K^*trans*^, TLG, and PET entropy were independent prognostic factors for RFS. We also identified K^*trans*^, V_*p*_, SUV, and entropy as independent predictors of OS. These findings indicate that PET- and MRI-derived functional parameters may have a different prognostic significance in patients with oropharyngeal and hypopharyngeal carcinoma. K^*trans*^estimates the time-dependent leakage from vessels into the interstitial tumor space. Higher K^*trans*^ values have been associated with better survival and more favorable treatment outcomes in patients with HNSCC.^[11]^ These results are consistent with our current findings and those from a previous study.^[22]^ Theoretically, vascular permeability should be inversely correlated with hypoxia, which is associated with a poor response to radiotherapy and unfavorable prognosis in patients with malignancies.^[42]^ Patients with high pretreatment K^*trans*^values are expected to have a more favorable survival. A better penetration of chemotherapeutic drugs into the tumor volume because of an increased permeability can also explain the improved survival observed in these patients.^[11]^ Herein, K_*ep*_ did not predict survival but V_*p*_ values were associated with a lower overall OS. K_*ep*_ is a rate constant denoting the transfer from the extravascular space to the plasma volume. Jasen et al^[43]^ reported reduced K_*ep*_ values in hypoxic lymph nodes of patients with HNSCC. V_*p*_—a measure of the intravascular volume or tumor vascularity—has been shown to predict treatment response in patients with spinal metastases.^[44]^

Uniformity and entropy are measures of imaging heterogeneity derived from the gray-level co-occurrence matrix. Here, we found that PET entropy values predicted both OS and RFS. In a study of patients with non-small cell lung cancer,^[35]^ entropy has been independently associated with OS as well. In contrast, PET uniformity has been associated with survival in patients with nasopharyngeal or oropharyngeal carcinoma.^[23,45]^ These results suggest that the significance of the pretreatment MRI- and PET-derived parameters for predicting survival in patients with HNSCC may depend on tumor subsite.

Some limitations of our study merit consideration. First, we used an SUV of 2.5 for tumor contouring. This method may not consistently delineate the exact tumor extent but is in line with most previous studies that have focused on PET parameters in patients with HNSCC^[19,23,46,47]^—ultimately allowing a direct comparison with the published literature. We attempted to use the adaptive threshold method for tumor segmentation,^[22]^ but the results did not differ appreciably from those using an SUV of 2.5 for tumor contouring. No single widely accepted tumor segmentation method in PET imaging currently exist.^[48]^ Furthermore, the values of DCE-MRI or DWI parameters are dependent on the selected ROI. Although manual contouring of the tumor is operator-dependent, ROIs in this study were drawn by an experienced head and neck radiologist in an effort to minimize potential biases. Finally, the limited sample size precluded cross-validation. Our findings need external validation in larger cohorts before more definitive conclusions could be drawn.

In summary, PET-derived TLG and the texture feature entropy were independent adverse prognostic factors for treatment response in patients with hypopharyngeal carcinoma undergoing CRT. Their combination enabled identification of prognostic subgroups at higher risk for treatment failure—thus allowing timely shifts in treatment strategies or salvage surgery. K^*trans*^—a DCE MRI parameter—was significant in predicting survival. Its combination with entropy and TLG allowed stratifying RFS, whereas its assessment alongside with entropy, SUV, and V*p* led to a better OS stratification compared with the traditional TNM staging system.

## Author contributions

**Conceptualization:** Sheng-Chieh Chan, Chih-Kai Wong, Shu-Hang Ng.

**Data curation:** Chih-Kai Wong.

**Formal analysis:** Sheng-Chieh Chan, Chih-Kai Wong, Nai-Ming Cheng.

**Funding acquisition:** Sheng-Chieh Chan.

**Project administration:** Sheng-Chieh Chan, Shu-Hang Ng.

**Writing – original draft:** Sheng-Chieh Chan, Chih-Kai Wong, Chia-Hsun Hsieh.

**Writing – review & rditing:** Sheng-Chieh Chan, Shu-Hang Ng, Tzu-Chen Yen, Chun-Ta Liao.

## Supplementary Material

Supplemental Digital Content

## References

[1] Spector JG Sessions DG Haughey BH Delayed regional metastases, distant metastases, and second primary malignancies in squamous cell carcinomas of the larynx and hypopharynx. Laryngoscope 2001;111:1079–87. 11404625 10.1097/00005537-200106000-00028

[2] Kuo P Sosa JA Burtness BA Treatment trends and survival effects of chemotherapy for hypopharyngeal cancer: analysis of the national cancer data base. Cancer 2016;122:1853–60. 27019213 10.1002/cncr.29962

[3] Allal AS Cancer of the pyriform sinus: trends towards conservative treatment. Bull Cancer 1997;84:757–62. 9339204

[4] Hall SF Groome PA Irish J The natural history of patients with squamous cell carcinoma of the hypopharynx. Laryngoscope 2008;118:1362–71. 18496152 10.1097/MLG.0b013e318173dc4a

[5] Nishimura H Sasaki R Yoshida K Radiotherapy for Stage I or II hypopharyngeal carcinoma. J Radiation Res 2012;53:892–9. 10.1093/jrr/rrs044 PMC3483846 22988283

[6] Jacobs MA Ibrahim TS Ouwerkerk R MR imaging: brief overview and emerging applications. Radiographics 2007;27:1213–29. 17620478 10.1148/rg.274065115

[7] Vogel DWT Thoeny HC Cross-sectional imaging in cancers of the head and neck: how we review and report. Cancer Imaging 2016;16:20 1–15. 27487932 10.1186/s40644-016-0075-3 PMC4971750

[8] Becker M Zaidi H Imaging in head and neck squamous cell carcinoma: the potential role of PET/MRI. Br J Radiol 2014;87:20130677 1–15. 24649835 10.1259/bjr.20130677 PMC4067029

[9] Metcalfe P Liney G Holloway L The potential for an enhanced role for MRI in radiation-therapy treatment planning. Technol Cancer Res Treat 2013;12:429–46. 23617289 10.7785/tcrt.2012.500342 PMC4527434

[10] Bernstein JM Homer JJ West CM Dynamic contrast-enhanced magnetic resonance imaging biomarkers in head and neck cancer: potential to guide treatment? A systematic review. Oral Oncol 2014;50:963–70. 25116700 10.1016/j.oraloncology.2014.07.011

[11] Chawla S Kim S Loevner L Prediction of disease-free survival in patients with squamous cell carcinomas of the head and neck using dynamic contrast-enhanced MR imaging. Am J Neuroradiol 2011;32:778–84. 21349969 10.3174/ajnr.A2376 PMC7965863

[12] Kim S Loevner LA Quon H Prediction of response to chemoradiation therapy in squamous cell carcinomas of the head and neck using dynamic contrast-enhanced MR imaging. AJNR Am J Neuroradiol 2010;31:262–8. 19797785 10.3174/ajnr.A1817 PMC7964131

[13] Ng SH Liao CT Lin CY Dynamic contrast-enhanced MRI, diffusion-weighted MRI and 18F-FDG PET/CT for the prediction of survival in oropharyngeal or hypopharyngeal squamous cell carcinoma treated with chemoradiation. Eur Radiol 2016;26:4162–72. 26911889 10.1007/s00330-016-4276-8

[14] Ng SH Lin CY Chan SC Dynamic contrast-enhanced MR imaging predicts local control in oropharyngeal or hypopharyngeal squamous cell carcinoma treated with chemoradiotherapy. PLoS One 2013;8:e72230 1–11. 23951300 10.1371/journal.pone.0072230 PMC3737151

[15] Driessen JP Kempen PM Heijden GJ Diffusion-weighted imaging in head and neck squamous cell carcinomas: a systematic review. Head Neck 2015;37:440–8. 24347513 10.1002/hed.23575

[16] Vansteenkiste JF PET scan in the staging of non-small cell lung cancer. Lung Cancer (Amsterdam, Netherlands) 2003;42:S27–37. 10.1016/s0169-5002(03)00302-7 14611912

[17] Schrevens L Lorent N Dooms C The role of PET scan in diagnosis, staging, and management of non-small cell lung cancer. Oncologist 2004;9:633–43. 15561807 10.1634/theoncologist.9-6-633

[18] Lim JS Yun MJ Kim M-J CT and PET in stomach cancer: preoperative staging and monitoring of response to therapy. Radiographics 2006;26:143–56. 16418249 10.1148/rg.261055078

[19] Pak K Cheon GJ Nam H-Y Prognostic value of metabolic tumor volume and total lesion glycolysis in head and neck cancer: a systematic review and meta-analysis. J Nucl Med 2014;55:884–90. 24752671 10.2967/jnumed.113.133801

[20] Koyasu S Nakamoto Y Kikuchi M Prognostic value of pretreatment 18F-FDG PET/CT parameters including visual evaluation in patients with head and neck squamous cell carcinoma. Am J Roentgenol 2014;202:851–8. 24660716 10.2214/AJR.13.11013

[21] Roh JL Kim JS Kang BC Clinical significance of pretreatment metabolic tumor volume and total lesion glycolysis in hypopharyngeal squamous cell carcinomas. J Surg Oncol 2014;110:869–75. 25088392 10.1002/jso.23729

[22] Chan SC Cheng NM Hsieh CH Multiparametric imaging using 18F-FDG PET/CT heterogeneity parameters and functional MRI techniques: prognostic significance in patients with primary advanced oropharyngeal or hypopharyngeal squamous cell carcinoma treated with chemoradiotherapy. Oncotarget 2017;8:62606–21. 28977973 10.18632/oncotarget.15904 PMC5617533

[23] Cheng NM Fang YH Chang JT Textural features of pretreatment 18F-FDG PET/CT images: prognostic significance in patients with advanced T-stage oropharyngeal squamous cell carcinoma. J Nucl Med 2013;54:1703–9. 24042030 10.2967/jnumed.112.119289

[24] Oh JS Kang BC Roh JL Intratumor textural heterogeneity on pretreatment (18)F-FDG PET images predicts response and survival after chemoradiotherapy for hypopharyngeal cancer. Ann Surg Oncol 2015;22:2746–54. 25487968 10.1245/s10434-014-4284-3

[25] Ng SH Lin CY Chan SC Clinical utility of multimodality imaging with dynamic contrast-enhanced MRI, diffusion-weighted MRI, and 18F-FDG PET/CT for the prediction of neck control in oropharyngeal or hypopharyngeal squamous cell carcinoma treated with chemoradiation. PLoS One 2014;9:e115933 1–19. 25531391 10.1371/journal.pone.0115933 PMC4274121

[26] Huang SH O'Sullivan B Overview of the 8th edition TNM classification for head and neck cancer. Curr Treat Options Oncol 2017;18:40 1–13. 28555375 10.1007/s11864-017-0484-y

[27] Wang HM Wang CS Chen JS Cisplatin, tegafur, and leucovorin: a moderately effective and minimally toxic outpatient neoadjuvant chemotherapy for locally advanced squamous cell carcinoma of the head and neck. Cancer 2002;94:2989–95. 12115388 10.1002/cncr.10570

[28] Tofts PS Brix G Buckley DL Estimating kinetic parameters from dynamic contrast-enhanced T(1)-weighted MRI of a diffusable tracer: standardized quantities and symbols. J Magn Reson Imaging 1999;10:223–32. 10508281 10.1002/(sici)1522-2586(199909)10:3<223::aid-jmri2>3.0.co;2-s

[29] Lin YC Chan TH Chi CY Blind estimation of the arterial input function in dynamic contrast-enhanced MRI using purity maximization. Magn Reson Med 2012;68:1439–49. 22383386 10.1002/mrm.24144

[30] Eisenhauer EA Therasse P Bogaerts J New response evaluation criteria in solid tumours: revised RECIST guideline (version 1.1). Eur J Cancer 2009;45:228–47. 19097774 10.1016/j.ejca.2008.10.026

[31] Hoffman HT Karnell LH Shah JP Hypopharyngeal cancer patient care evaluation. Laryngoscope 1997;107:1005–17. 9260999 10.1097/00005537-199708000-00001

[32] Goel R Moore W Sumer B Clinical practice in PET/CT for the management of head and neck squamous cell cancer. AJR Am J Roentgenol 2017;209:289–303. 28731808 10.2214/AJR.17.18301

[33] Lim R Eaton A Lee NY 18F-FDG PET/CT metabolic tumor volume and total lesion glycolysis predict outcome in oropharyngeal squamous cell carcinoma. J Nucl Med 2012;53:1506–13. 22895812 10.2967/jnumed.111.101402

[34] Hatt M Tixier F Pierce L Characterization of PET/CT images using texture analysis: the past, the present... any future. Eur J Nucl Med Mol Imaging 2017;44:151–65. 27271051 10.1007/s00259-016-3427-0 PMC5283691

[35] Cook GJ O’Brien ME Siddique M Non-small cell lung cancer treated with erlotinib: heterogeneity of F-FDG uptake at PET-association with treatment response and prognosis. Radiology 2015;276:883–93. 25897473 10.1148/radiol.2015141309

[36] Tixier F Le Rest CC Hatt M Intratumor heterogeneity characterized by textural features on baseline 18F-FDG PET images predicts response to concomitant radiochemotherapy in esophageal cancer. J Nucl Med 2011;52:369–78. 21321270 10.2967/jnumed.110.082404 PMC3789272

[37] Cohen EE Karrison TG Kocherginsky M Phase III randomized trial of induction chemotherapy in patients with N2 or N3 locally advanced head and neck cancer. J Clin Oncol 2014;32:2735–43. 25049329 10.1200/JCO.2013.54.6309 PMC4876357

[38] Haddad R O’Neill A Rabinowits G Induction chemotherapy followed by concurrent chemoradiotherapy (sequential chemoradiotherapy) versus concurrent chemoradiotherapy alone in locally advanced head and neck cancer (PARADIGM): a randomised phase 3 trial. Lancet Oncol 2013;14:257–64. 23414589 10.1016/S1470-2045(13)70011-1

[39] Hitt R Grau JJ Lopez-Pousa A A randomized phase III trial comparing induction chemotherapy followed by chemoradiotherapy versus chemoradiotherapy alone as treatment of unresectable head and neck cancer. Ann Oncol 2014;25:216–25. 24256848 10.1093/annonc/mdt461

[40] Chapman CH Parvathaneni U Yom SS Revisiting induction chemotherapy before radiotherapy for head and neck cancer, part I: carcinoma of non-nasopharyngeal sites. Future Oncol 2017;13:469–75. 28169592 10.2217/fon-2016-0502

[41] Kelly JR Husain ZA Burtness B Treatment de-intensification strategies for head and neck cancer. Eur J Cancer 2016;68:125–33. 27755996 10.1016/j.ejca.2016.09.006 PMC5734050

[42] Gueulette J Octave-Prignot M De Costera BM Intestinal crypt regeneration in mice: a biological system for quality assurance in non-conventional radiation therapy. Radiother Oncol 2004;73:S148–54. 15971332 10.1016/s0167-8140(04)80038-0

[43] Jansen JF Schoder H Lee NY Noninvasive assessment of tumor microenvironment using dynamic contrast-enhanced magnetic resonance imaging and 18F-fluoromisonidazole positron emission tomography imaging in neck nodal metastases. Int J Radiat Oncol Biol Phys 2010;77:1403–10. 19906496 10.1016/j.ijrobp.2009.07.009 PMC2888682

[44] Chu S Karimi S Peck KK Measurement of blood perfusion in spinal metastases with dynamic contrast-enhanced magnetic resonance imaging: evaluation of tumor response to radiation therapy. Spine (Phila Pa 1976) 2013;38:E1418–24. 23873238 10.1097/BRS.0b013e3182a40838 PMC5757658

[45] Chan SC Chang KP Fang YD Tumor heterogeneity measured on F-18 fluorodeoxyglucose positron emission tomography/computed tomography combined with plasma Epstein-Barr Virus load predicts prognosis in patients with primary nasopharyngeal carcinoma. Laryngoscope 2017;127:E22–8. 27435352 10.1002/lary.26172

[46] Seol YM Kwon BR Song MK Measurement of tumor volume by PET to evaluate prognosis in patients with head and neck cancer treated by chemo-radiation therapy. Acta Oncol 2010;49:201–8. 20100156 10.3109/02841860903440270

[47] Xie P Yue JB Zhao HX Prognostic value of 18F-FDG PET-CT metabolic index for nasopharyngeal carcinoma. J Cancer Res Clin Oncol 2010;136:883–9. 19936788 10.1007/s00432-009-0729-7 PMC11828335

[48] Zaidi H El Naqa I PET-guided delineation of radiation therapy treatment volumes: a survey of image segmentation techniques. Eur J Nucl Med Mol Imaging 2010;37:2165–87. 20336455 10.1007/s00259-010-1423-3

