# Supplementary material for: Textural features on 18F-FDG PET/CT and dynamic contrast-enhanced MR imaging for predicting treatment response and survival of patients with hypopharyngeal carcinoma
Source: Medicine (Baltimore). 2019 Aug 16;98(33):e16608. doi: 10.1097/MD.0000000000016608 (PMC6831375; doi:10.1097/MD.0000000000016608)
Supplement: Supplemental Digital Content [file medi-98-e16608-s001.doc]

**Supplementary table 1**

**Functional MRI and PET parameters used in this study.**

| **Parameter** | **Mean ± SD** | **Range** |
| --- | --- | --- |
| **Functional MRI parameters** |  |  |
| K*^trans^* | 0.486 ± 0.369 | 0.010–1.580 |
| V*_e_* | 0.203 ± 0.157 | 0.003–0.883 |
| K*_ep_* | 3.231 ± 2.714 | 0.157–13.325 |
| V*_p_**1000 | 0.047 ± 0.112 | 0–0.635 |
| ADC | 1020.520 ± 169.996 | 764–1567.7 |
| **Conventional PET parameters** |  |  |
| SUVmax | 15.192 ± 5.469 | 4.107–30.500 |
| MTV | 19.315 ± 17.139 | 1.234–85.430 |
| TLG | 136.142 ± 136.932 | 4.378-738.550 |
| **PET heterogeneity parameters** |  |  |
| **NGTDM**  Coarseness | 0.020 ± 0.015 | 0.004–0.075 |
| Contrast | 0.076 ± 0.444 | 0–3.462 |
| Busyness | 0.199 ± 0.156 | 0.024–0.726 |
| Complexity | 60.122 ± 81.587 | 2.032–539.066 |
| **NGLCM**  Uniformity | 0.003 ± 0.003 | 0.001–0.021 |
| Entropy | 6.426 ± 0.720 | 3.871–7.217 |
| Homogeneity | 0.186 ± 0.041 | 0.066–0.316 |
| Dissimilarity | 9.892 ± 3.237 | 4.114–24.542 |
| Inverse difference moment | 0.103 ± 0.035 | 0.010–0.227 |
| MRI: magnetic resonance imaging, PET: positron emission tomography, SD: standard deviation, K*^trans^*: volume transfer rate constant, V*_e_*: relative extravascular extracellular space, K*_ep_*: efflux rate constant, V*_p_*: relative vascular plasma volume, ADC: apparent diffusion coefficient, SUVmax: maximum standardized uptake value, MTV: metabolic tumor volume, TLG: total lesion glycolysis, NGLCM: normalized gray-level co-occurrence matrix, NGTDM: neighborhood gray-tone difference matrix. | | |

| **Supplementary table 2**  **Comparison of overall survival and recurrence-free survival according to the scoring stratification system.** | | | | | | | |
| --- | --- | --- | --- | --- | --- | --- | --- |
| **Overall survival** | | |  | **Recurrence-free survival** | | |  |
| **Score** | ***P* value** | **HR** |  | **Score** | ***P* value** | **HR** |  |
| **0** (1/11)* | Reference | Reference |  | **0** (6/18) | Reference | Reference |  |
| **1** (3/19) | .628 | 1.927 |  | **1** (14/28) | .123 | 2.057 |  |
| **2** (11/22) | .025 | 7.205 |  | **2** (9/12) | .002 | 4.985 |  |
| **3–4** (7/9) | .002 | 19.896 |  | **3** (3/3) | < .0001 | 10.166 |  |

*****Event/patient number**,** HR: hazard ratio
